# Phosphorylation of AQP4 by LRRK2 R1441G impairs glymphatic clearance of IFNγ and aggravates dopaminergic neurodegeneration

**DOI:** 10.1038/s41531-024-00643-z

**Published:** 2024-01-31

**Authors:** Heng Huang, Lishan Lin, Tengteng Wu, Cheng Wu, Leping Zhou, Ge Li, Fengjuan Su, Fengyin Liang, Wenyuan Guo, Weineng Chen, Qiuhong Jiang, Yalun Guan, Xuejiao Li, Pingyi Xu, Yu Zhang, Wanli Smith, Zhong Pei

**Affiliations:** 1 Department of Neurology, The First Affiliated Hospital, Sun Yat-sen University, Guangdong Provincial Key Laboratory of Diagnosis and Treatment of Major Neurological Diseases, National Key Clinical Department and Key Discipline of Neurology, Guangzhou, China; 2 Department of Neurology, The First Affiliated Hospital of Guangzhou Medical University, Guangzhou, China; 3 Guangdong Cardiovascular Institute, Guangdong Provincial People’s Hospital, Guangdong Academy of Medical Sciences, Guangzhou, China; 4 Department of Neurology, The Third Affiliated Hospital of Guangzhou Medical University, Guangzhou, China; 5 Guangdong Provincial Key Laboratory of Laboratory Animals, Guangdong Laboratory Animals Monitoring Institute, Guangzhou, China; 6 Department of Psychiatry, Division of Neurobiology, Johns Hopkins University School of Medicine, Baltimore, MD 21287 USA

**Keywords:** Parkinson's disease, Parkinson's disease

## Abstract

Aquaporin-4 (AQP4) is essential for normal functioning of the brain’s glymphatic system. Impaired glymphatic function is associated with neuroinflammation. Recent clinical evidence suggests the involvement of glymphatic dysfunction in *LRRK2*-associated Parkinson’s disease (PD); however, the precise mechanism remains unclear. The pro-inflammatory cytokine interferon (IFN) γ interacts with LRRK2 to induce neuroinflammation. Therefore, we examined the AQP4-dependent glymphatic system’s role in IFNγ-mediated neuroinflammation in *LRRK2*-associated PD. We found that LRRK2 interacts with and phosphorylates AQP4 in vitro and in vivo. AQP4 phosphorylation by *LRRK2 R1441G* induced AQP4 depolarization and disrupted glymphatic IFNγ clearance. Exogeneous IFNγ significantly increased astrocyte expression of IFNγ receptor, amplified AQP4 depolarization, and exacerbated neuroinflammation in *R1441G* transgenic mice. Conversely, inhibiting LRRK2 restored AQP4 polarity, improved glymphatic function, and reduced IFNγ-mediated neuroinflammation and dopaminergic neurodegeneration. Our findings establish a link between LRRK2-mediated AQP4 phosphorylation and IFNγ-mediated neuroinflammation in *LRRK2*-associated PD, guiding the development of LRRK2 targeting therapy.

## Introduction

Parkinson’s disease (PD) is a common chronic progressive neurodegenerative disease, with the main pathological characteristics of dopaminergic (DA) neuron loss in the substantia nigra and the accumulation of Lewy bodies in the brain^1^. Although genetic PD accounts for only a small proportion of all cases, studies on genetic PD have substantially deepened our comprehension of the molecular mechanisms underlying PD^2^. The *LRRK2* mutation is the most common genetic cause of PD, and its variants are associated with a high risk of sporadic PD^3^. However, despite intensive research for more than 15 years, the precise role of *LRRK2* in PD remains largely elusive.

LRRK2 is a large protein with functional GTPase and kinase domains. Therefore, LRRK2 possess both GTPase and kinase activities^4^. GTPases function as molecular switches cycling between inactive GDP-bound and active GTP-bound state^5^. In the active GTP-bound state, GTPases activate kinase. LRRK2 can bind to hydrolyze GTP via ROC domain to regulate kinase activity^6^. Mutations in either GTPase or kinase domain cause late-onset PD, suggesting that both GTPase and kinase activities are involved in the development of PD^7^. However, it is still inconsistent whether mutations in the ROC-COR domain increase kinase activity. In addition, the most common mutation *G2019S* has been shown to induce neurotoxic effects in a kinase-dependent manner without altering GTPase activity^8^. Therefore, how altered GTPase activity link to neurodegeneration is still uncertain.

Neurodegenerative diseases, including PD, have been associated with impaired functioning of the glymphatic system, a pathway responsible for clearing waste from the brain^9,10^. Aquaporin-4 (AQP4) is mainly localized to the perivascular astrocytic end-feet^11^, where it maintains brain homeostasis through the glymphatic system, which involves parenchymal waste clearance and immune regulation^12,13^. Recently, AQP4-mediated glymphatic impairment has been implicated in PD. For example, elevated expression of AQP4 has been observed in the neocortex of PD brains^14^, and loss of AQP4 reportedly induces PD-like pathology in animals^15^. Similarly, patients with *LRRK2*-associated PD have been found to exhibit considerable enlargement of the perivascular space (PVS), a key component of the glymphatic system^16^, suggesting that dysfunction of the glymphatic system is also involved in *LRRK2*-associated PD. However, the exact mechanism underlying the association between the glymphatic system and *LRRK2*-associated PD remains unclear.

Mounting evidence indicates that immune dysregulation may serve as a significant factor in initiating PD pathology, considering that it can be detected before the onset of PD^17^. LRRK2 exhibits significant expression within immune cells, contributing to the modulation of immune-related disorders^18^. Thus, mutant *LRRK2* has been proposed to interact with immune insults to induce nigral cell loss^19^. However, how immune dysregulation contributes to DA degeneration in *LRRK2*-associated PD remains unelucidated. Interferon γ (IFNγ), a proinflammatory cytokine primarily secreted by immune cells^20^, reportedly mediates neuroinflammation and DA degeneration in PD^21^. In *LRRK2*-associated PD, IFNγ interacts with mutant *LRRK2*, triggering neuroinflammation and DA degeneration^22^. Interestingly, glymphatic dysfunction has been implicated in neuroinflammation. For example, systemic injection of bacterial endotoxin lipopolysaccharides (LPS) can disrupt the glymphatic system^23^ which is responsible for clearing pro-inflammatory cytokines as brain waste^24^. Thus, when the glymphatic system fails, pro-inflammatory cytokines become trapped within the brain, consequently accelerating neuroinflammation.

In this study, we aimed to explore the role of the glymphatic system in *LRRK2*-associated PD. Our finding indicates that *LRRK2 R1441G* disrupts the glymphatic system by directly phosphorylating and depolarizing AQP4. Additionally, we demonstrate that the glymphatic system plays a vital role in clearing IFNγ, whereas LRRK2-induced impairment of glymphatic functions leads to IFNγ accumulation, exacerbating neuroinflammation and neurotoxicity. Furthermore, we found that neuroinflammation resulting from glymphatic impairment was dependent on LRRK2, as these detrimental effects were abolished by administrating an LRRK2 inhibitor. This highlights the involvement of the glymphatic system in *LRRK2*-associated neuroinflammation.

## Results

### *LRRK2 R1441G* impairs glymphatic system clearance and decreases AQP4 polarity

**Fig. 1:**
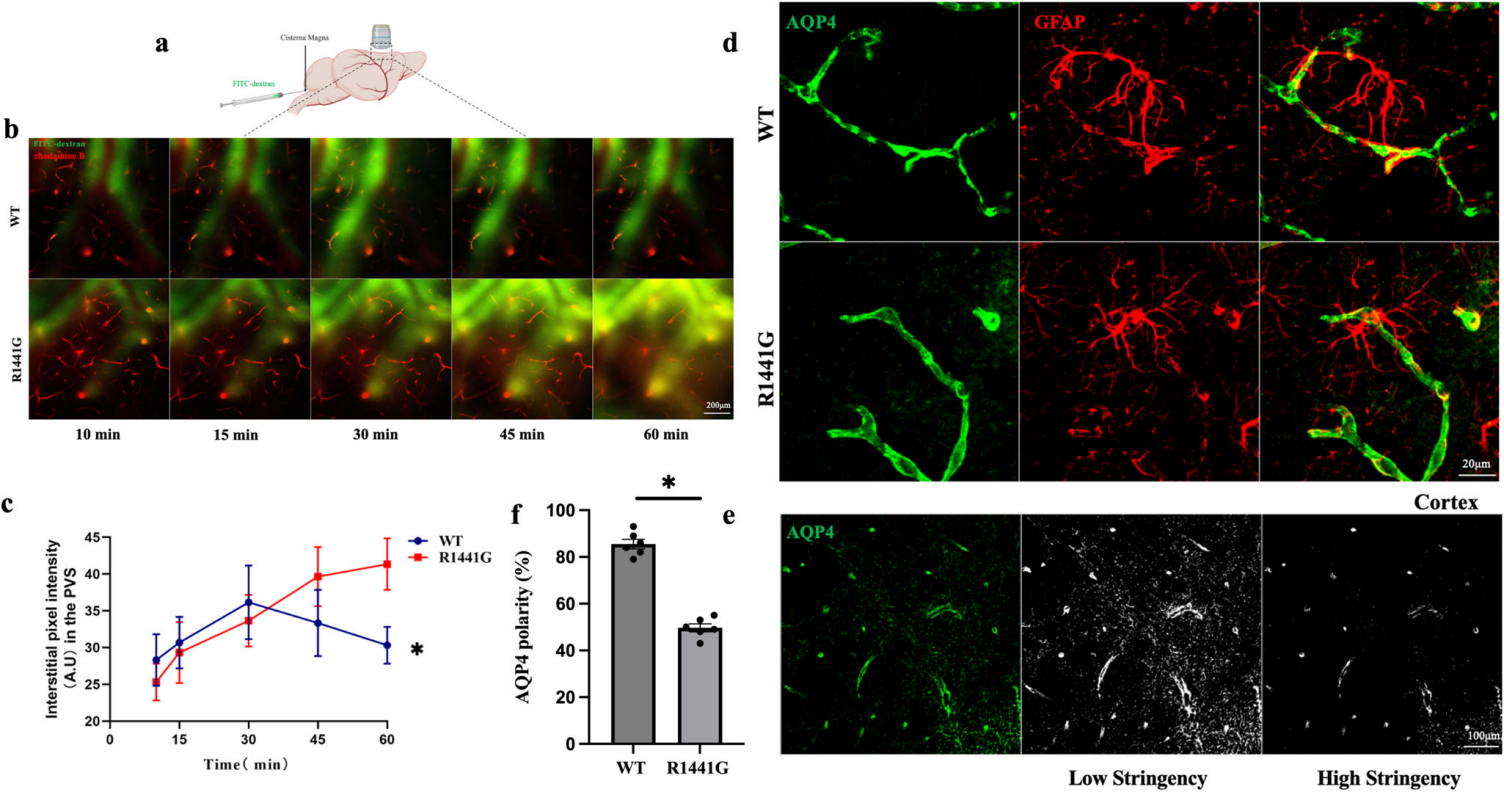
*LRRK2 R1441G* impairs glymphatic system clearance. **a** Schema showing the intracisternal injection of fluorescein isothiocyanate (FITC)-dextran for in vivo two-photon imaging. **b** Representative image of FITC-dextran along the perivascular space (PVS) penetrating the brain parenchyma of *LRRK2* WT and *R1441G* mice, 100 μm below the cortical surface (scale bar = 200 μm). **c** Statistic analysis of the fluorescence intensity of FITC-dextran in the PVS shown in (**b**). Datasets are expressed as means ± standard error of mean. *n* = 6 per group. *p < 0.05. Two-way ANOVA and Tukey’s post hoc test were used for analysis. **d** Representative immunostaining of AQP4 and GFAP in the *LRRK2* WT and *R1441G* mice (scale bar = 20 μm). **e** Schematic diagram illustrating the calculation process for AQP4 polarity (scale bar = 100 μm). **f** Quantification of (**d**). *LRRK2 R1441G* mutant led to a decrease in perivascular AQP4 polarity compared to *LRRK2* WT group. Datasets are expressed as means ± standard error of mean. *n* = 6 per group. *p < 0.05. Independent-sample t-tests were used for analysis.

The glymphatic system plays a crucial role in clearing abnormally accumulated proteins in the brain^25^. To explore the clearance function of the glymphatic system in *LRRK2* wild type (WT) and *LRRK2 R1441G (R1441G)* transgenic mice, fluorescein isothiocyanate [FITC]-dextran and rhodamine B were used as tracers of cerebrospinal fluid (CSF) and blood vessels (Fig. 1a**)**. Two-photon in vivo imaging was employed to measure the clearance of FITC-dextran through the glymphatic system.In WT mice, FITC tracer appeared in the paravascular space 10 min after the injection. The fluorescence intensity increased by 15 min, peaked at 30 min, began decreasing at 45 min, and decreased further at 60 min. In contrast, while FITC-dextran was also detected in the paravascular space of *R1441G* mice after 10 min, the fluorescence intensity continuously increased at 15, 30, 45, and 60 min (Fig. 1b, c). These findings suggest that *LRRK2 R1441G* mutation impairs the clearance function of the glymphatic system.

AQP4 polarity plays a crucial role in facilitating the influx of subarachnoid CSF and the efflux of interstitial fluid (ISF) solutes to the brain parenchyma^26^, which maintains the normal function of the glymphatic system^11^. Under physiological conditions, AQP4 expression is predominantly polarized to the perivascular astrocytic endfeet. However, in certain disease conditions, the expression of AQP4 shifts from the endfeet to the soma^27^. AQP4 immunofluorescence was localized in the astrocytic endfeet in WT mice. Conversely, in the *R1441G* group, AQP4 was depolarized and observed in the astrocytic soma (Fig. 1d). As previously elucidated, AQP4 polarity was quantified as the ratio of the low-stringency area to the high-stringency area^28^. The low-stringency threshold encompasses the overall area of AQP4 immunoreactivity, while the high-stringency threshold corresponds to the area of AQP4 immunoreactivity in perivascular end feet (Fig. 1e). Calculation of AQP4 polarity further revealed significantly lower values in the *R1441G* group compared to the WT group (Fig. 1f). Taken together, *R1441G* mutation decreased AQP4 polarity and impaired glymphatic system clearance.

### LRRK2 interacts with AQP4, and mutant *LRRK2* regulates AQP4 polarity through phosphorylation

**Fig. 2:**
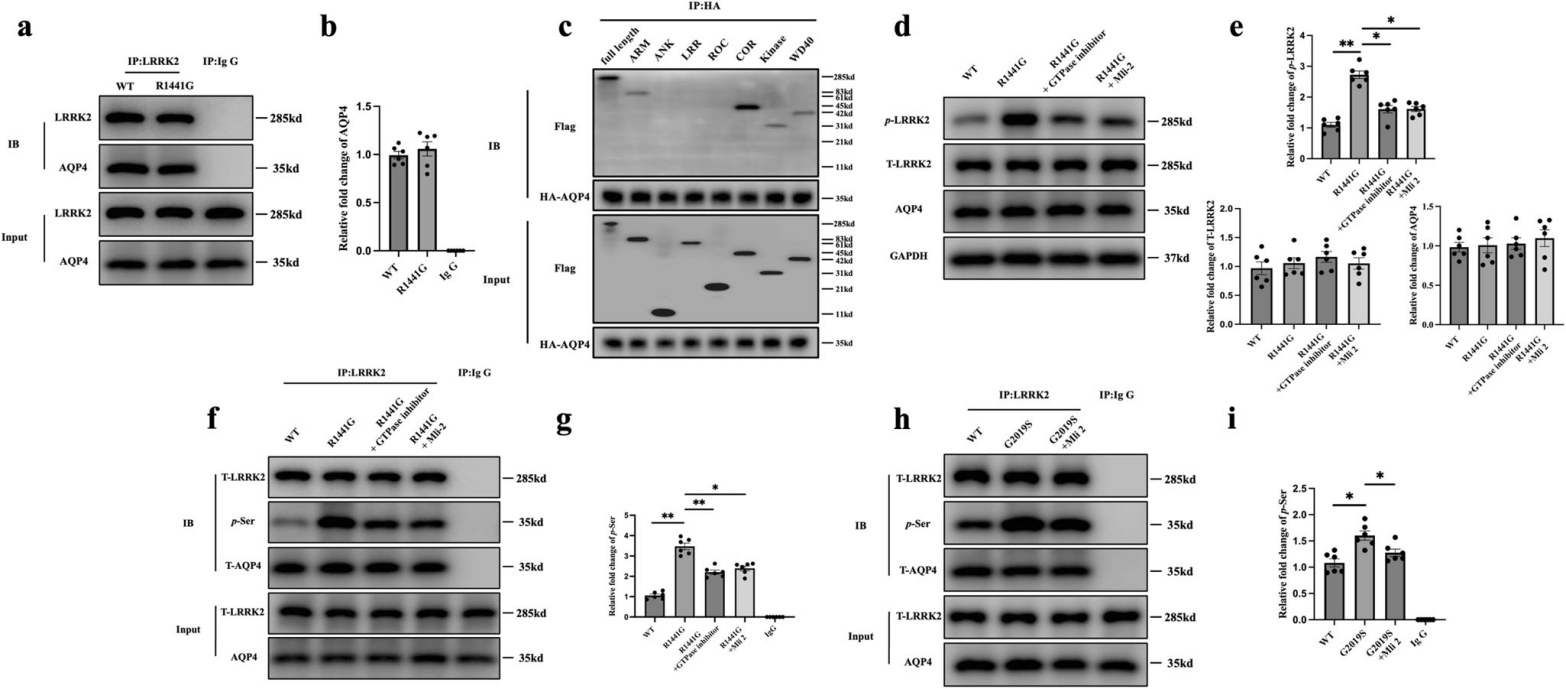
LRRK2 interacts with AQP4 and phosphorylates AQP4. **a** Immunoblotting of proteins from brain lysates of *LRRK2* WT and *R1441G* transgenic mice subjected to co-immunoprecipitation (co-IP) by an antibody against LRRK2. **b** Quantification of (**a**). Datasets are expressed as means ± standard error of mean. *n* = 6 per group. One-way ANOVA and Tukey’s post hoc test were used for analysis. **c** Immunoblot of proteins from HeLa cell lysates transfected with FLAG-LRRK2 different domains and HA-AQP4, subjected to co-IP by antibody to HA. **d** Immunoblotting of proteins from brain lysates of the *LRRK2* WT*, LRRK2 R1441G*, *LRRK2 R1441G*+GTPase inhibitor and *LRRK2 R1441G*+Mli-2 groups. **e** Quantification of (**d**). Datasets are expressed as means ± standard error of mean. *n* = 6 per group. *p < 0.05; **p < 0.01. Two-way ANOVA and Tukey’s post hoc test were used for analysis. **f** Immunoblotting of phosphorylated serine (*p*-Ser) in AQP4 pulled down by LRRK2 from brain lysates of the *LRRK2* WT*, LRRK2 R1441G*, *LRRK2 R1441G*+GTPase inhibitor and *LRRK2 R1441G*+Mli-2 groups. **g** Quantification of (**f**). Datasets are expressed as means ± standard error of mean. *n* = 6 per group. *p < 0.05; **p < 0.01. Two-way ANOVA and Tukey’s post hoc test were used for analysis. **h** Immunoblot of proteins from HeLa cell lysates transfected with FLAG-LRRK2 WT/*G2019S* and HA-AQP4, subjected to co-IP by antibody to LRRK2. **i** Quantification of (**h**). Datasets are expressed as means ± standard error of mean. *n* = 6 per group. *p < 0.05. Two-way ANOVA and Tukey’s post hoc test were used for analysis.

To investigate how the *R1441G* mutant affects the polarity of AQP4, we conducted the co-immunoprecipitation (co-IP) experiment to examine whether LRRK2 interacts with AQP4. LRRK2 successfully pulled down AQP4 using brain lysates from both WT and *R1441G* mice. However, no substantial difference was seen in the amount of AQP4 pulled down by LRRK2, indicating that the *R1441G* mutation may not affect the ability of LRRK2 to bind to AQP4 (Fig. 2a, b). To explore which domain of LRRK2 bind with AQP4, we co-transfected the different domains of LRRK2 with AQP4. AQP4 could pull down ARM, COR, Kinase and WD40 domain, indicating that AQP4 interacted LRRK2 through several different domains (Fig. 2c). Furthermore, western blot studies showed that there was no difference of total AQP4 protein level between the WT and *R1441G* mouse brains with or without GTPase inhibitor or kinase inhibitor treatment (Fig. 2d, e, Supplementary Fig. 1a, b).

The polarity of AQP4 depends on its overall protein expression and recruitment from intracellular stores to the plasma membrane, known as membrane compartmentalization^29^, the latter of which is significantly influenced by AQP4 phosphorylation^30^. Since the phosphorylation sites of AQP4 are mainly serine (Ser)^30,31^, an anti-LRRK2 antibody was used to pull down AQP4 in the brain tissues from WT and *R1441G* transgenic mice, and then Ser phosphorylation of AQP4 (*p*-AQP4) was detected. We observed considerably higher levels of *p*-AQP4 in *R1441G* mice than in WT mice. Importantly, this effect was partially reversed by LRRK2 inhibitors, indicating that *R1441G* mutation increases AQP4 phosphorylation (Fig. 2f, g, Supplementary Fig. 1c, d).

To investigate whether the impact of mutant *LRRK2* on AQP4 polarity is specific to the *R1441G* mutation or attributable to increased LRRK2 phosphorylation, we co-transfected *LRRK2 G2019S* with AQP4. The co-IP results revealed that the *G2019S* mutation led to an elevated expression of *p*-AQP4 whereas Mli-2 treatment mitigated *G2019S*-induced *p*-AQP4 (Fig. 2h, i). In summary, these findings indicate that the *LRRK2 G2019S* mutation could also phosphorylate AQP4, underscoring the broader impact of *LRRK2* mutations on AQP4 dynamics.

**Fig. 3:**
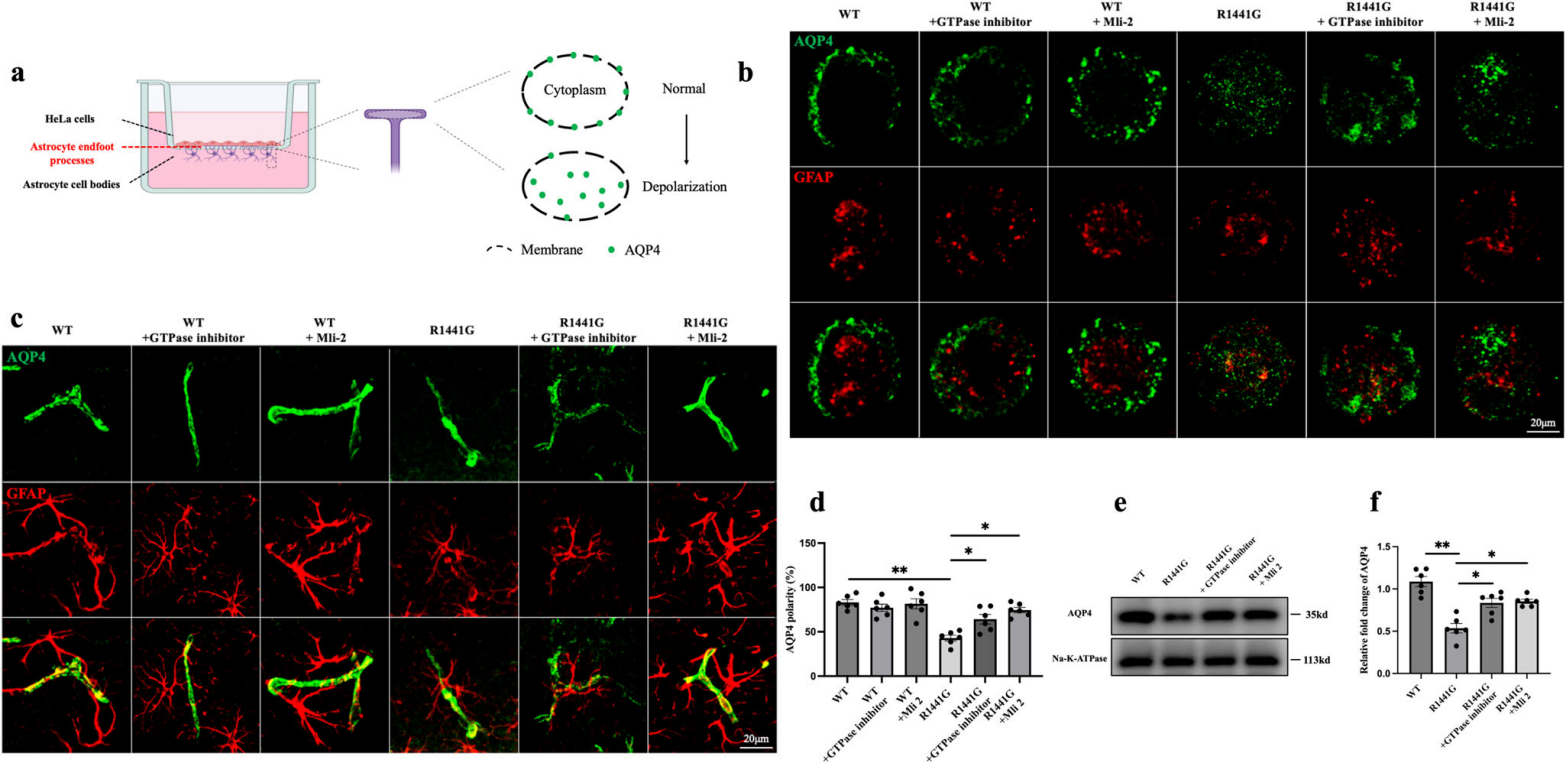
LRRK2 regulates AQP4 polarity. **a** HeLa cells (red) are seeded on the top and primary culture of astrocytes (purple) is seeded on the bottom of the filter. **b** Representative images of the membrane compartmentalization of AQP4 at the endfeet processes of primary astrocytes from the *LRRK2* WT and *R1441G* transgenic mice treated with or without a GTPase inhibitor/Mli-2 groups (scale bar = 20 μm). **c** Representative images of AQP4 polarity in the *LRRK2* WT and *R1441G* transgenic mice treated with or without a GTPase inhibitor/Mli-2 groups (scale bar = 20 μm). **d** Quantification of (**c**). Datasets are expressed as means ± standard error of mean. *n* = 6 per group. *p < 0.05; **p < 0.01. Two-way ANOVA and Tukey’s post hoc test were used for analysis. **e** Immunoblotting of membrane proteins from brain lysates of *LRRK2* WT and *R1441G* transgenic mice treated with or without a GTPase inhibitor/Mli-2. **f** Quantification of (**e**). Datasets are expressed as means ± standard error of mean. *n* = 6 per group. *p < 0.05; **p < 0.01. Two-way ANOVA and Tukey’s post hoc test were used for analysis.

The depolarizing effect of the *R1441G* mutation on AQP4 was further examined in an in vitro transwell system. Primary astrocytes isolated from WT or *R1441G* mice were seeded in the bottom wells. The endfeet of the astrocytes could pass through the filter pores to the upper wells, establishing close connections with HeLa cells (Fig. 3a). Glial Fibrillary Acidic Protein (GFAP), the most commonly used astrocytic marker was used to co-stain with AQP4 to evaluate AQP4 polarity. As depicted in Fig. 3b, AQP4 predominantly expressed on the surface membrane of astrocytic endfeet in the WT group. Conversely, in the *R1441G* group, AQP4 was internalized into the cytoplasm. Treatment with either GTPase inhibitor(Fx2149) or kinase inhibitor (Mli-2) successfully restored the altered distribution of AQP4 in *R1441G* astrocytes. This observation suggests that *LRRK2 R1441G* modifies AQP4 polarity through phosphorylation, and the restoration by LRRK2 inhibitors indicates potential therapeutic intervention for this modification.

To examine whether LRRK2 inhibitors can also rescue the AQP4 polarity in vivo, we administrated both GTPase inhibitor and kinase inhibitor to the WT and *R1441G* mice. LRRK2 inhibitors can significantly decrease AQP4 depolarization induce by *R1441G* mutation, indicating by AQP4 immunoactivity in endfeet of astrocyte, but not soma (Fig. 3c, d). Furthermore, Western blot study showed that LRRK2 inhibitors also alleviate the decreased expression of AQP4 in membrane caused by *R1441G* mutation (Fig. 3e, f, Supplementary Fig. 1e, f). The similar results were also observed with *G2019S* mutation (Supplementary Fig. 1g, h).These results indicated that LRRK2 inhibitors can improve AQP4 polarity. Collectively, these findings provide evidence that mutant *LRRK2* affects AQP4 polarity through phosphorylation, which may impair the clearance function of the glymphatic system.

### IFNγ is involved in LPS-induced neuroinflammation in mutant *LRRK2* mice

**Fig. 4:**
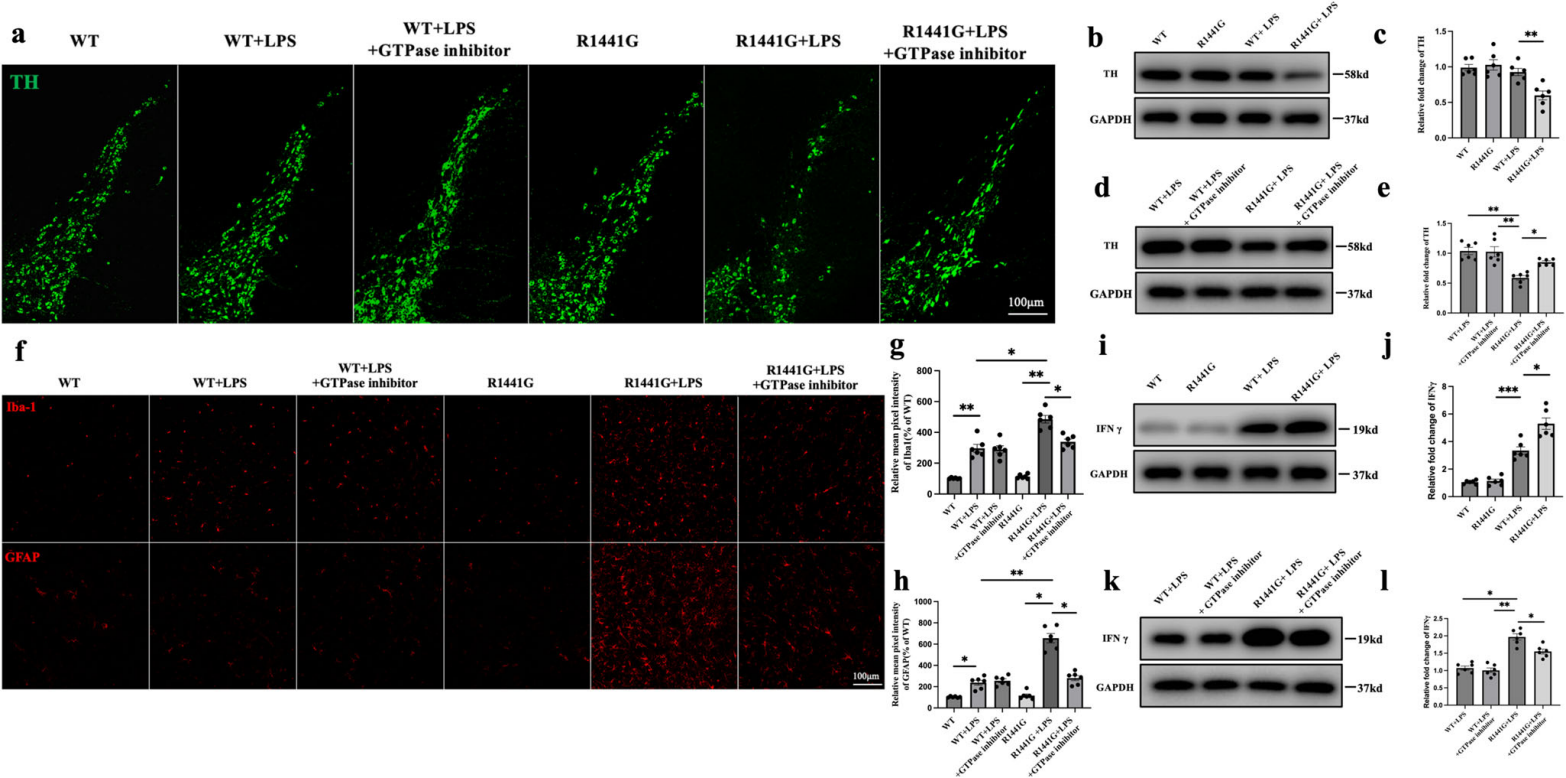
IFNγ is involved in LPS-induced neuroinflammation in mutant *LRRK2* mice. **a** Representative images of tyrosine hydroxylase (TH) in the control and experimental groups (scale bar = 100 μm). **b** Immunoblotting of TH from midbrain lysates of *LRRK2* WT and *R1441G* transgenic mice treated with or without lipopolysaccharide (LPS). **c** Quantification of (**b**). Datasets are expressed as means ± standard error of mean. *n* = 6 per group. **p < 0.01. Two-way ANOVA and Tukey’s post hoc test were used for analysis. **d** Immunoblotting of TH from midbrain lysates of *LRRK2* WT and *R1441G* mice treated with or without a GTPase inhibitor/LPS. **e** Quantification of (**d**). Datasets are expressed as means ± standard error of mean. *n* = 6 per group. *p < 0.05; **p < 0.01. Two-way ANOVA and Tukey’s post hoc test were used for analysis. **f** Representative images of Iba**-**1 and GFAP in the control and experimental groups (scale bar = 100 μm). **g** Quantification of Iba-1 in (**f**). Datasets are expressed as means ± standard error of mean. *n* = 6 per group. *p < 0.05; **p < 0.01. Two-way ANOVA and Tukey’s post hoc test were used for analysis. **h** Quantification of GFAP in (**f**). Datasets are expressed as means ± standard error of mean. *n* = 6 per group. *p < 0.05; **p < 0.01. Two-way ANOVA and Tukey’s post hoc test were used for analysis. **i** Immunoblotting of IFNγ from brain lysates of *LRRK2* WT and *R1441G* transgenic mice treated with or without LPS. **j** Quantification of (**i**). Datasets are expressed as means ± standard error of mean. *n* = 6 per group. *p < 0.05; ***p < 0.001. Two-way ANOVA and Tukey’s post hoc test were used for analysis. **k** Immunoblotting of IFNγ from brain lysates of *LRRK2* WT and *R1441G* mice treated with or without a GTPase inhibitor/LPS. **l** Quantification of (**k**). Datasets are expressed as means ± standard error of mean. *n* = 6 per group. *p < 0.05; **p < 0.01. Two-way ANOVA and Tukey’s post hoc test were used for analysis.

LPS reportedly induces neuroinflammation and DA degeneration in mutant *LRRK2* mice^32^. Immunofluorescence staining results showed that there was significant loss of tyrosine hydroxylase (TH) neurons in *R1441G* + LPS group compared with WT, WT + LPS and *R1441G* groups (Fig. 4a). Furthermore, the protein levels of TH in midbrain also decreased in the *R1441G* + LPS group (Fig. 4b, c). Similarly,the *R1441G* group showed no microgliosis and astrogliosis compared with the WT group. However, the reactivity of both microglia and astrocytes was more remarkable in the *R1441G* + LPS group than in the WT + LPS group (Fig. 4f–h). Furthermore, treatment with LRRK2 inhibitor rescued the loss of TH neurons and mitigated gliosis in the *R1441G* + LPS group, while there were no difference between WT + LPS group with or without inhibitor treatment. (Fig. 4a–h).

IFNγ has also been implicated in DA neurodegeneration^33^. Therefore, IFNγ levels were examined. We found that the protein levels of IFNγ were markedly elevated in both WT and *R1441G* mice after LPS treatment. Notably, the *R1441G* + LPS group exhibited much higher IFNγ levels compared to the WT + LPS group (Fig. 4i, j). The administration of LRRK2 inhibitor successfully reduced the elevated levels of IFNγ (Fig. 4k, l).

Collectively, these findings suggest that the interaction between *LRRK2 R1441G* and LPS synergistically triggers the activation of inflammatory cells and the loss of DA neurons, with IFNγ playing a role in LPS-induced neuroinflammation.

### *LRRK2 R1441G* impairs the clearance of IFNγ through the glymphatic system

**Fig. 5:**
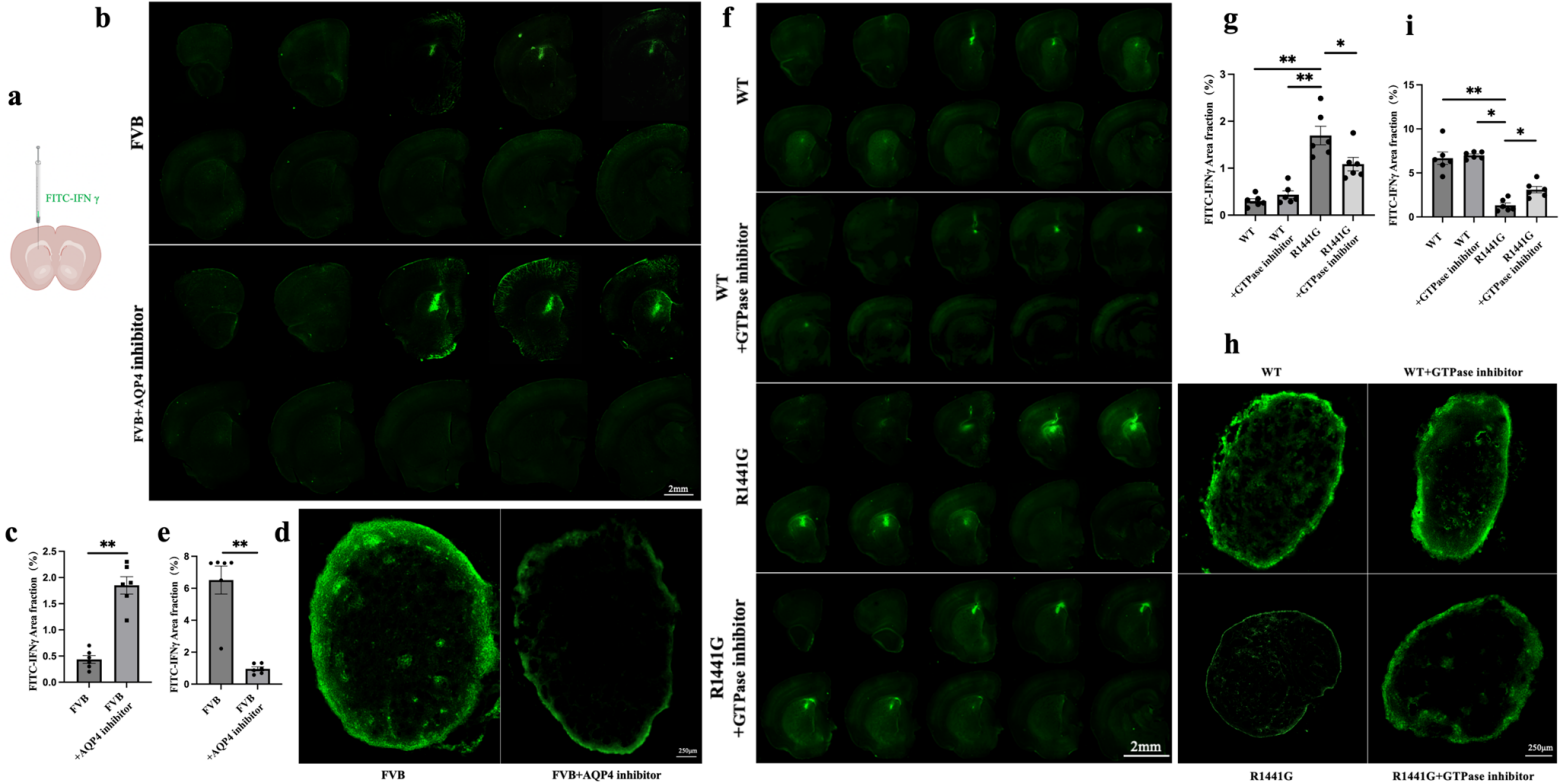
*LRRK2 R1441G* impairs the clearance of IFNγ through the glymphatic system. **a** Schematic of Intrastriatum injection of fluorescein isothiocyanate (FITC)-IFNγ (anteroposterior, +1.5 mm; mediolateral, −1.5 mm; and dorsoventral, +2.5 mm from the bregma). **b** Representative images of brain sections showing the coverage of FITC-IFNγ in the parenchyma of FVB mice treated with or without an AQP4 inhibitor(scale bar = 2 mm). **c** Quantification of (**b**). Datasets are expressed as means ± standard error of mean. *n* = 6 per group. **p < 0.01. Independent-sample t-tests were used for analysis. **d** Representative images of deep cervical lymph nodes (dCLNs) showing the fluorescence intensity of the FITC-IFNγ(scale bar = 250 μm). **e** Quantification of (**d**). Datasets are expressed as means ± standard error of mean. *n* = 6 per group. **p < 0.01. Independent-sample t-tests were used for analysis. **f** Representative images of brain sections showing FITC-IFNγ coverage in the brain parenchyma of *LRRK2* WT and *R1441G* mice treated with or without a GTPase inhibitor(scale bar = 2 mm). **g** Quantification of (**f**). Datasets are expressed as means ± standard error of mean. *n* = 6 per group. *p < 0.05; **p < 0.01. Two-way ANOVA and Tukey’s post hoc test were used for analysis. **h** Representative images showing the FITC-IFNγ drainage into the dCLNs(scale bar = 250 μm). **i** Quantification of (**h**). Datasets are expressed as means ± standard error of mean. *n* = 6 per group. *p < 0.05; **p < 0.01. Two-way ANOVA and Tukey’s post hoc test were used for analysis.

To investigate whether IFNγ can be cleared from the brain via the AQP4-dependent glymphatic system, FITC-IFNγ was injected into the striatum of FVB mice (background strain for both *LRRK2* WT and *R1441G* transgenic mice) treated with or without an AQP4 inhibitor (Fig. 5a). The fluorescence intensity in the brain parenchyma was measured to assess the efflux of FITC-IFNγ 2 h after the intraparenchymal injection. A notably higher dye residual in the parenchyma was observed in the FVB mice treated with the AQP4 inhibitor than in those not treated with the inhibitor (Fig. 5b, c). Previous studies have indicated that macromolecules present in the parenchyma or ISF primarily drain into deep cervical lymph nodes (dCLNs) following efflux^34^. Remarkably, compared with control animals, animals treated with an AQP4 inhibitor displayed significantly reduced fluorescence intensity in the dCLNs (Fig. 5d, e). These findings indicate that IFNγ is cleared from the brain tissue via the AQP4-dependent glymphatic system.

Next, the efflux of IFNγ from the brain parenchyma was examined to determine whether it is affected by the *R1441G* mutation. Two hours after injection, FITC-IFNγ was found to be significantly higher in the parenchyma of *R1441G* mice than in WT mice (Fig. 5f, g). Similarly, the fluorescence intensity in the dCLNs exhibited a significant reduction in the *R1441G* group (Fig. 5h, i). These effects were reversed by treatment with a LRRK2 inhibitor (Fig. 5f–i). There was no difference in fluorescence intensity between WT mice treated with or without inhibitors. These findings suggest that the *LRRK2 R1441G* mutation impairs the clearance of IFNγ through the glymphatic system.

### IFNγ upregultes the expression of IFNγ receptor-2 and LRRK2 in *LRRK2 R1441G* mice, further aggravating the loss of AQP4 polarity

Binding of IFNγ to its receptors (IFNGR) induces the activation of receptor-associated downstream signaling factors^35^. IFNGR-1 and -2 are two subunits of functional IFNGR^36^. While IFNGR-1 is essential for ligand binding, it cannot induce a biological response independently. IFNGR-2 is associated with IFNGR-1 in an IFNγ-dependent manner and plays a vital role in the transmission of functional responses^37^. Therefore, we focused on the transduction of IFNGR-2 to determine the impact of IFNγ on LRRK2 and AQP4 in the brain.

**Fig. 6:**
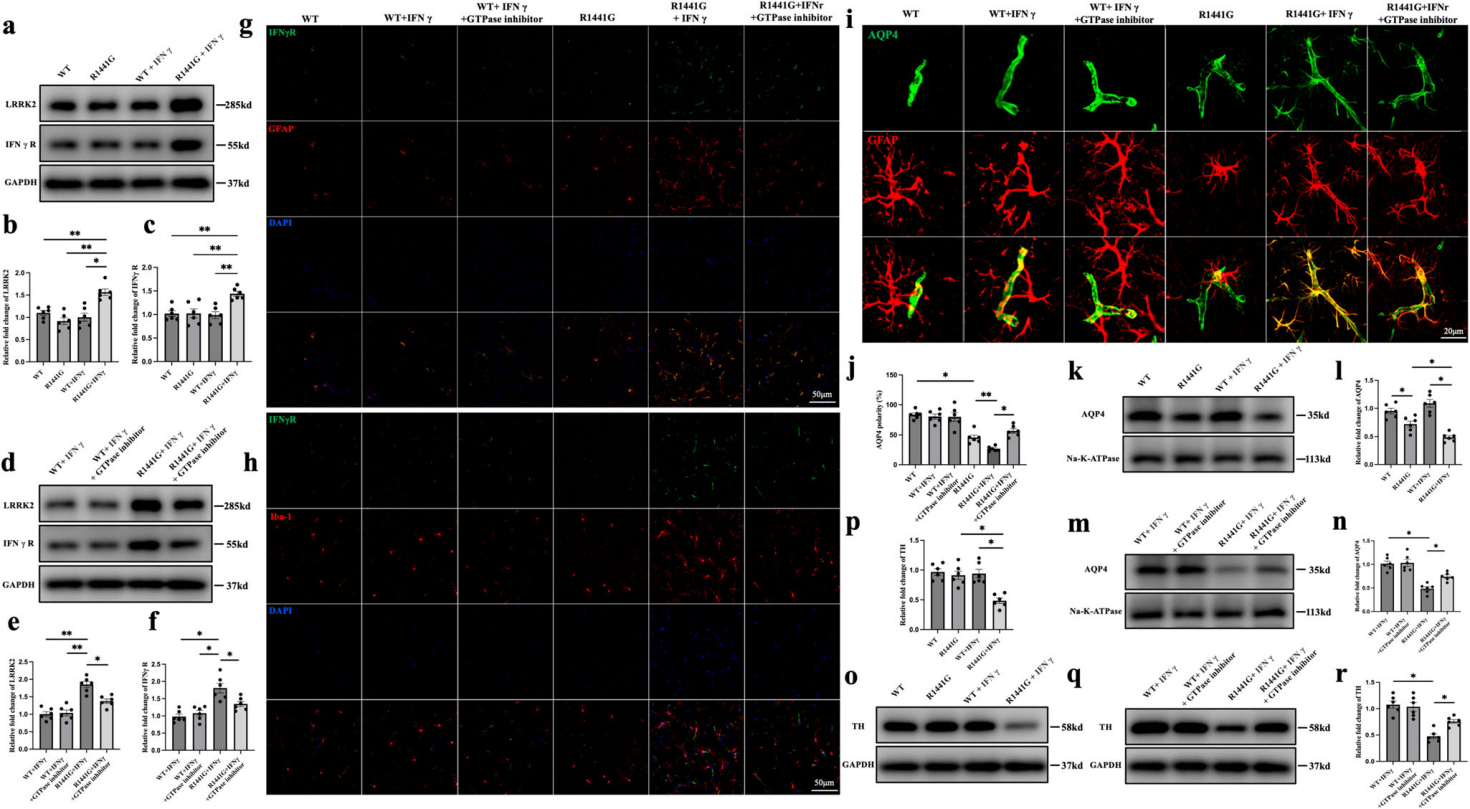
IFNγ upregulates IFNGR-2 expression in astrocytes, increases LRRK2 expression, and reduces AQP4 polarity in *LRRK2 R1441G* mice. **a** Immunoblotting of LRRK2 and IFNγR from brain lysates of *LRRK2* WT and *R1441G* transgenic mice treated with or without IFNγ. **b** Quantification of LRRK2 in (**a**). Datasets are expressed as means ± standard error of mean. *n* = 6 per group. *p < 0.05; **p < 0.01. Two-way ANOVA and Tukey’s post hoc test were used for analysis. **c** Quantification of IFNγR in (**a**). Datasets are expressed as means ± standard error of mean. *n* = 6 per group. **p < 0.01. Two-way ANOVA and Tukey’s post hoc test were used for analysis. **d** Immunoblotting of LRRK2 and IFNγR from brain lysates of *LRRK2* WT and *R1441G* transgenic mice treated with or without IFNγ and a GTPase inhibitor. **e** Quantification of LRRK2 in (**d**). Datasets are expressed as means ± standard error of mean. *n* = 6 per group. *p < 0.05; **p < 0.01. Two-way ANOVA and Tukey’s post hoc test were used for analysis. **f** Quantification of IFNγR in (**d**). Datasets are expressed as means ± standard error of mean. *n* = 6 per group. *p < 0.05. Two-way ANOVA and Tukey’s post hoc test were used for analysis. **g** Representative images of IFNγR and GFAP in the control and experimental groups (scale bar = 50 μm). **h** Representative images of IFNγR and Iba-1 in the control and experimental groups (scale bar = 50 μm). **i** Representative images of AQP4 polarity in the control and experimental groups (scale bar = 20 μm). **j** Quantification of (**i**). Datasets are expressed as means ± standard error of mean. *n* = 6 per group. *p < 0.05; **p < 0.01. Two-way ANOVA and Tukey’s post hoc test were used for analysis. **k** Immunoblotting of membrane AQP4 from brain lysates of *LRRK2* WT and *R1441G* transgenic mice treated with or without IFNγ. **l** Quantification of (**k**). Datasets are expressed as means ± standard error of mean. *n* = 6 per group. *p < 0.05. Two-way ANOVA and Tukey’s post hoc test were used for analysis. **m** Immunoblotting of membrane AQP4 from brain lysates of *LRRK2* WT and *R1441G* transgenic mice treated with or without IFNγ and a GTPase inhibitor. **n** Quantification of (**m**). Datasets are expressed as means ± standard error of mean. *n* = 6 per group. *p < 0.05. Two-way ANOVA and Tukey’s post hoc test were used for analysis. **o** Immunoblotting of TH from brain lysates of *LRRK2* WT and *R1441G* transgenic mice treated with or without IFNγ. **p** Quantification of (**o**). Datasets are expressed as means ± standard error of mean. *n* = 6 per group. *p < 0.05. Two-way ANOVA and Tukey’s post hoc test were used for analysis. **q** Immunoblotting of TH from brain lysates of *LRRK2* WT and *R1441G* transgenic mice treated with or without IFNγ and a GTPase inhibitor. **r** Quantification of (**q**). Datasets are expressed as means ± standard error of mean. *n* = 6 per group. *p < 0.05. Two-way ANOVA and Tukey’s post hoc test were used for analysis.

To that end, IFNγ was injected into the striatum of WT and *LRRK2 R1441G* mice, and the expression of IFNGR-2 was compared 1 week later. Markedly increased expressions of both IFNGR-2 and LRRK2 were observed in the *R1441G* mice but not in the WT mice after IFNγ treatment (Fig, 6a–c). Notably, treatment with a LRRK2 inhibitor partially downregulated the expression of both IFNGR-2 and LRRK2 in the *R1441G* + IFNγ group (Fig. 6d–f). Furthermore, both reactive astrocyte and microglia were evident. However, IFNGR-2 mainly co-localized with GFAP but not with Iba-1 in the brain of *R1441G* mice, indicating that IFNGR-2 was mainly expressed in astrocytes and LRRK2 inhibitor could partially reverse the effects (Fig. 6g, h). Next, we assessed the polarity of AQP4 after the IFNγ treatment. AQP4 depolarization was obvious after IFNγ treatment in the *R1441G* mice, as evidenced by the increase in immunoactivity of AQP4 in the soma of astrocyte and the decrease of AQP4 polarity, while this effect could be rescued by LRRK2 inhibitor (Fig. 6i, j). Furthermore, western blotting study showed that AQP4 expression in the cell membrane was lower in the *R1441G* + IFNγ group than in the *R1441G* group. However, treatment with LRRK2 inhibitor partially restored the loss of AQP4 polarity (Fig. 6k–n). IFNγ treatment also decreased the expression of TH in *R1441G* mice, while LRRK2 inhibitor could rescue it (Fig. 6o–r). There was no significant difference in the expression of TH in the WT mice treated with or without IFNγ or LRRK2 inhibitor (Fig. 6o–r). These findings suggest that IFNγ upregulates the expression of IFNGR-2 in astrocytes, reduces AQP4 polarity and induces TH neuron loss.

## Discussion

Our study provides evidence that mutant *LRRK2* directly phosphorylates AQP4, thereby impairing the AQP4-dependent glymphatic system. Additionally, we found that the AQP4-dependent glymphatic system facilitates the removal of IFNγ from the brain. However, dysfunction of the AQP4-dependent glymphatic system caused by mutant *LRRK2* diminished the clearance of IFNγ. Consequently, IFNγ accumulated in the brain tissue, resulting in neuroinflammation and DA neurodegeneration. Interestingly, administration of an LRRK2 inhibitor effectively rescued the AQP4-dependent glymphatic system impairment induced by mutant *LRRK2*, thereby attenuating IFNγ-associated neuroinflammation and DA neurodegeneration.

The present study has clinical implications. In clinical practice, glymphatic system dysfunction can be detected using MRI^34^. Thus, LRRK2 inhibitors may be beneficial to patients with glymphatic system dysfunction, as they can repair glymphatic system dysfunction and protect against neuroinflammation-mediated DA neurodegeneration. Glymphatic dysfunction is a shared pathway in various neurodegenerative diseases, including *LRRK2*-associated PD^16^. Congruently, we found that the glymphatic system was impaired in *LRRK2 R1441G* mice. Furthermore, we found that LRRK2 interacts with AQP4, a key molecule in the glymphatic system, whereas phosphorylation of AQP4 by mutant *LRRK2* is associated with glymphatic impairment. In contrast, inhibition of AQP4 phosphorylation rescued the *LRRK2*-induced glymphatic impairment, suggesting that mutant *LRRK2* impairs glymphatic function through AQP4 phosphorylation.

The polarity expression of AQP4 is essential for the functioning of the glymphatic system^27^. AQP4 is normally expressed at the endfeet of astrocytes in a highly polarized manner, while the loss of polarity of AQP4 is associated with glymphatic dysfunction^38^. In the present study, the membrane expression of AQP4 was reduced, and AQP4 was moved from the endfeet to the cytoplasm in *LRRK2 R1441G* astrocytes, suggesting that the loss of polarity of AQP4 is responsible for *LRRK2 R1441G*-induced glymphatic dysfunction. It is not clear how the *LRRK2 R1441G* mutant affects AQP4 polarity. However, inhibition of the *LRRK2 R1441G* mutant reduced phosphorylated AQP4 and revised AQP4 polarity, indicating that phosphorylation of AQP4 is involved in mutant *LRRK2*-mediated AQP4 depolarization. It is well documented that LRRK2 possess both GTPase and kinase activities^4^. Mutations in the ROC-COR domain have been reported to impair GTPase activity, suggesting an important role of GTPase activity in the development of PD^39^. However, it is still inconsistent whether mutations in the ROC-COR domain increase kinase activity. How altered GTPase activity link to neurodegeneration remains elusive. *R1441G* mutation are located within ROC-COR domain and has been reported to reduce GTPase activity which in turn indirectly increases LRRK2 kinase activity^40^. Consistently, either GTPase inhibitor or kinase inhibitor could attenuate *R1441G*-mediated AQP4 depolarization, suggesting that Kinase is indeed involved in *R1441G*-mediated phosphorylation of AQP4.

Regulation of AQP4 polarity involves intracellular trafficking through the endosome system^41^. Interestingly, phosphorylation and dephosphorylation play a substantial role in cellular trafficking of proteins, enabling their internalization into endosomes from the cell surface. For example, previous studies have shown that phosphorylation of the Ser residue in AQP2, another member of the AQP family, can influence its interaction with different endosomes within the endosome system, leading to changes in its intracellular location^42^. Similarly, AQP4 contains multiple phosphorylation sites^43^, prompting the hypothesis that the *LRRK2 R1441G* mutation may mediate AQP4 polarity distribution through AQP4 phosphorylation. Indeed, the *LRRK2 R1441G* mutation disrupted AQP4 polarity, reducing its expression in the cell membrane through phosphorylation. Conversely, inhibiting LRRK2 activity attenuated the depolarization induced by the *LRRK2 R1441G* mutation. To gain deeper insights into the regulation of AQP4 polarity, further investigations are warranted to explore the relationship between AQP4 and the endosome system. These investigations may unveil novel insights into the regulation of AQP4 polarity and its implications in various cellular processes.

Immune dysfunction and neuroinflammation contribute to the initiation and progression of PD^44^. IFNγ plays a key role in mediating glial reactions and inducing neuroinflammation in PD^21^. Under normal conditions, IFNγ levels are very low and undetectable in the brain. However, during neuroinflammation, the disruption of the blood–brain barrier and T-cell infiltration can lead to a substantial increase in IFNγ levels in the brain^45^. Recent evidence highlights the crucial role of the glymphatic system in regulating neuroinflammation^24^. Indeed, the glymphatic system has demonstrated its capacity to clear various pro-inflammatory cytokines, including interleukin-1β and tumor necrosis factor-α, from the brain^24,46^. Accordingly, we investigated the potential involvement of the glymphatic system in clearing IFNγ from the brain. We found that the injected FITC-labeled IFNγ considerably accumulated in the parenchyma of the mice treated with an AQP4 inhibitor, suggesting that IFNγ is cleared from the brain via the AQP4-dependent glymphatic system. This discovery highlights the importance of the glymphatic system in controlling neuroinflammation by facilitating the clearance of IFNγ, which has implications for understanding and treating PD and other neuroinflammatory conditions.

Previous research has highlighted the connection between IFNγ and mutant *LRRK2* in bridging neuroinflammation and DA degeneration in *LRRK2*-PD^22,32^. IFNγ induces neuroinflammation and DA neurodegeneration primarily through IFNγ receptor (IFNGR)-dependent canonical signaling^47^. In fact, animals lacking IFNGR are reportedly completely unresponsive to IFNγ stimulation, indicating the crucial role of IFNGR in mediating its effects^33^. In this study, we found that IFNγ upregulated the protein level of LRRK2 and increased the expression of IFNGR-2 in astrocytes markedly while mutant *LRRK2* mice treated with IFNγ. This was accompanied by depolarization of AQP4, indicated by the translocation of AQP4 from astrocytic endfeet to cell bodies. Importantly, inhibiting LRRK2 partially reversed IFNγ-induced AQP4 depolarization, underscoring the critical role of LRRK2 activity in this process. Notably, *LRRK2* is a target gene of IFNγ and is regulated by its activity^48^. Once IFNγ binds to IFNGR, it activates the JAK-STAT pathway^35^, which in turn induces LRRK2 expression. This suggests that IFNγ may interact with IFNGR in astrocytes, activate LRRK2, and cause glymphatic dysfunction through AQP4 depolarization. Subsequently, glymphatic dysfunction leads to the accumulation of IFNγ, exacerbating the dysfunction, thereby establishing a vicious circle. Consequently, the interaction between the *R1441G* mutation and IFNγ impairs the glymphatic system, increasing neuroinflammation and contributing to DA neurodegeneration in a synergistic manner. Interestingly, glymphatic impairment can be reversed by inhibiting LRRK2, suggesting that targeting LRRK2 activity might break the vicious cycle. Notably, IFNγ might also induce neuroinflammation and neurotoxicity in other cell types^33^. In the present study, we were unable to detect IFNGR-2 expression in microglia, suggesting that IFNγ alone is insufficient to induce microglial IFNGR-2 expression. Other studies have reported that IFNγ requires additional inflammatory stimulators, such as LPS, to activate microglia^49^. However, IFNγ reportedly increases the expression of major histocompatibility complex (MHC)-I in neurons and MHC-I/II in microglia^33^. Further investigations are needed to explore the specific contributions of different cell types in *LRRK2*-associated PD.

In the present study, we conducted co-IP experiments to identify the LRRK2 domains responsible for the interaction with AQP4. We found that several domains including ARM, COR, Kinase and WD40 interacted with AQP4 with different bind affinities whereas ANK, LRR and ROC failed to interact with AQP4. Given that *R1441G* is located within ROC domain, this observation may further support that *R1441G* mutation did not affect the interaction of LRRK2 with AQP4. It is puzzling that *G2019S* did not affect the interaction because *G2019S* is located in kinase domain. One possible explanation may be that *G2019S* does not disrupt the domain structure responsible for interacting with AQP4. However, it should be noted that our Co-IP results are based on overexpression which may result in non-physiological artifacts. Therefore, it should be cautious to interpret the interactions between WT and *LRRK2* mutations with AQP4. Interestingly, COR domain had the strongest interaction with AQP4. It is generally believed that COR domain has an essential role for LRRK2 dimerization and regulation of kinase activity. Currently, 15 reported *LRRK2* variants including *Y1699C* mutation are located within COR domain^50^. Whether those *LRRK2* variants within COR domain can affect the interaction with AQP4 should be explored in the future study.

Protein-protein interactions are becoming attractive new drug target in drug discovery. In the present study, we found that the interaction of mutant *LRRK2* with AQP4 induced glymphatic impairment which further led to subsequent neuroinflammation and neurodegeneration, whereas a general GTPase inhibitor could attenuate those phenotypes. In the future, development of novel inhibitors specifically targeting the interaction of LRRK2 with AQP4 is desirable through mass spectrometry or peptide screening approaches.

In our current study, we have demonstrated the substantial role of the interaction between LRRK2 and AQP4 in the pathophysiology of *LRRK2*-associated PD. Mutant *LRRK2* was found to phosphorylate and depolarize AQP4, resulting in impairment of the glymphatic system. Additionally, this glymphatic dysfunction led to reduced clearance of IFNγ. As a result, the accumulation of IFNγ exacerbated *LRRK2*-induced neuroinflammation. Importantly, we found that inhibiting LRRK2 with an LRRK2 inhibitor could break this harmful cycle, effectively reducing neuroinflammation-related DA neurodegeneration. As glymphatic system dysfunction can be clinically assessed through neuroimaging, this finding holds great promise for the development of precise therapeutic approaches for PD.

Our study has several limitations. The transgenic mice utilized in our current study were designed to overexpress either WT *LRRK2* or the mutant *R1441G LRRK2*. It is essential to acknowledge that this experimental approach may introduce some artificial outcomes, particularly in the co-IP results. This is one potential limitation when comparing WT and mutant since both can bind to AQP4. Therefore, the findings from our co-IP experiments should be validated in the assays under physiological condition in future study.

In summary, our study sheds light on the critical link between LRRK2 and AQP4 in *LRRK2*-associated PD pathology. By understanding this mechanism, we can open new avenues for targeted therapies to tackle neuroinflammation and DA neurodegeneration.

## Methods

### Animals and LRRK2 inhibitors treatment

Animal procedures were conducted in accordance with the Guide for the Care and Use of Laboratory Animals of Sun Yat-sen University (Guangzhou, China). The animals were housed in a facility maintained under specific-pathogen-free (SPF) conditions and were provided with unlimited food and water. FVB/N-Khdrbs2Tg (LRRK2*R1441G)135Cjli/J (Strain #:009604) mice harbor a mutated form of the human leucine-rich repeat kinase 2 (*LRRK2 R1441G*) gene. To serve as wild-type (WT) controls, FVB/N-Tg (*LRRK2*)1Cjli/J (Strain #: 009610) mice expressing the WT human *LRRK2* gene were utilized. Both the WT and *R1441G LRRK2* mice were bred in the FVB background. For the experiments involving interferon-gamma (IFNγ) clearance, FVB mice (Strain #: 001800) were employed. All three types of mice were ordered from the Jackson Laboratory and bred at the Guangdong.

Three- and four-month-old WT or *R1441G* mice were subjected to intraperitoneal injections of either 0.9% sterile NaCl or LPS at a dose of 5 mg/kg (L2630, Sigma, USA) to induce a PD-like mouse model. For LRRK2 inhibitor treatment, GTPase inhibitor (Fx2149) was administered to mice at 10 mg/kg intraperitoneally, twice daily for three days, while kinase inhibitor (Mli-2) was administered at 10 mg/kg intraperitoneally, twice daily for ten days. Seven days after the LPS treatment, the mice were sacrificed for subsequent pathological studies. All experimental procedures were conducted in strict adherence to The Code of Ethics of the World Medical Association (Declaration of Helsinki) for animal experiments, ensuring the ethical treatment and welfare of the animals involved.

### DNA constructs and CO-IP assays

Different domains of LRRK2 tagged with FLAG were constructed and then co-transfected with HA-AQP4 in HeLa cells. Cell lysates or brain tissues from mice were homogenized in protein lysis buffer (Beyotime, Shanghai, China) for quantitation of total protein and immunoprecipitation assays. The Dynabeads protein G immunoprecipitation kit (Invitrogen, Carlsbad, CA) was employed for immunoprecipitation. Briefly, 1.5 mg of Dynabeads magnetic beads were incubated with 2 μg of LRRK2-specific antibody or immunoglobulin (Ig) G for 10 min at RT to bind to the antibody. The beads were then washed and incubated with 500 μL of brain lysis buffer overnight at 4 °C to form the magnetic bead–antibody-antigen complexes. The complexes were then washed thrice, and proteins were eluted with 50 μL of elution buffer from the beads and processed for subsequent functional assays.

### Primary culture of astrocytes

Newborn (1–2 days) *LRRK2* WT*/R1441G* transgenic mice were employed to isolate primary cortical astrocytes. The protocol was modified by McCarthy and de Vellis. Briefly, the conical tubes were pre-filled with 2 mL fresh complete medium (high glucose Dulbecco’s modified Eagle’s medium +10% fetal bovine serum + 100 U/mL penicillin–streptomycin), and then the brain cortex was collected after decapitation and removal of meninges. After mechanical dissociation, samples were passaged through 70-μm nylon cell strainers (BD Biosciences). Then, the undifferentiated mouse brain cells were seeded onto 75-cm^2^ flasks pre-coated with poly-L-lysine (Sigma-Aldrich) and 15 mL fresh medium was added. The cells were placed in a 37 °C incubator with a humidified atmosphere containing 5% CO_2_. The medium was replaced every other day. To detach the microglial and precursor cells, the flasks were shaken for 2 min each time when we changed the medium during the first week. GFAP immunostaining was used to detect the purity; over 95% of the cells were astrocytes.

### Co-culture on filter/cell culture inserts

The primary astrocytes were seeded onto the bottom of cell culture inserts (BD Biosciences) with a 1-μm pores (12 × 10^4^ cells/cm^2^) once the cells reached confluence. Subsequently, they were placed in an incubator for 6 h to facilitate cell attachment to the filter. The inserts were placed in a six-well plate filled with fresh medium. After astrocytes reached 80% confluence, which took about 7 days, HeLa cells were placed on the top of the filters (2 × 10^4^ cells/cm^2^)^29^. After 7–10 days, the HeLa cells established a monolayer, and then the cells were fixed with 4% formaldehyde.

### In vivo two-photon imaging of glymphatic pathway clearance

A total of 12 *LRRK2* WT and *R1441G* mice (*n* = 6 for each group) were used in this study. The mice were anesthetized with 1% pentobarbital and subsequently positioned in a stable stereotaxic device. The right parietal cortex was made at lambdoid suture coordinates: mediolateral (ML) 2.0 mm, anteroposterior (AP) 1.7 mm. For imaging purposes, a slender cranial window measuring 3 mm in diameter was created.

To evaluate the PVS–ISF exchange, 10 μL of 1% FITC-dextran in artificial CSF was intracisternally injected at a rate of 1 μL/min using a microsyringe (BASi, West Lafayette, IN). Immediately before imaging, 0.2 mL of rhodamine B (1% in saline; Sigma-Aldrich) was administered via tail injection to label the vasculature. A laser scanning microscope (Leica Microsystems, Wetzlar, Germany) was used for our study. The laser was operated at 800 nm, and 25× water-immersion objective lens was utilized for imaging. To analyze the clearance of FITC-dextran from brain parenchyma, lateral images up to 100–300 μm below the cortical surface of the *xyz* stacks (512 × 512 pixels, 2-μm resolution) were collected at a series of time points: 10, 15, 30, 45 and 60 min after the injection.

### Intraparenchymal injection of FITC-dextran

To evaluate the impact of mutant *LRRK2* on the interstitial solute drainage from the brain into dCLNs, *LRRK2* transgenetic mice were intraparenchymally injected with FITC-dextran. 1 μL of 1% FITC-dextran in artificial CSF was stereotactically injected into the parenchyma at a rate of 0.1 μL/min over 10 min with a syringe pump from the bregma of the brain(coordinates: AP 1.5 mm; ML 1.5 mm and dorsal/ventral −2.5 mm). The needle was held at the site for 10 min after the infusion was complete to avoid backflow. At 2 h after injection, dCLNs and brains were removed and fixed in 4% paraformaldehyde. Then, brains were cut into 50 μm-thick coronal brain slices via a vibrating blade microtome (Leica), while dCLNs(30 μm-thick) were sectioned using a freezing microtome (Leica). Images were acquired with a Leica confocal microscope and analyzed by a blinded investigator to evaluate the differences of glymphatic clearance between the control and experimental groups.

### Immunofluorescence

Brain sections were taken from −80 °C and kept at room temperature (RT) for 1 h to recover. The tissues were first washed with phosphate-buffered saline and then blocked for 1 h at RT. Then, brain sections were incubated with different primary antibodies overnight at 4 °C and secondary antibody for 1 h at RT, with essential washing in the interval. The following antibodies were used: LRRK2 (MJFF2 [c41–2]; Abcam, Cambridge, UK), AQP4 (Santa Cruz Biotechnology, Dallas, TX), Alexa 488- or 555-conjugated secondary antibodies (Cell Signaling Technology, Danvers, MA). Images were captured by a Nikon microscope (Tokyo, Japan). The polarity of AQP4 in each image was characterized by the ratio of low-stringency areas (the total area exhibiting AQP4 immunoreactivity) to high-stringency areas (the region with pronounced AQP4 immunoreactivity).

### Western blotting

Protein samples were prepared from the homogenized lysates of mouse brain tissue, and the concentrations were detected with the Pierc BCA Protein Assay Kit (Invitrogen). Proteins were separated by SDS-PAGE gel (Epizyme, China), transferred to a polyvinylidene fluoride membrane, and blocked with 5% milk for 1 h at RT. The membranes were then incubated with the following primary antibodies: LRRK2 (Abcam), AQP4 (Proteintect), *p*-Ser (Millipore), and GAPDH (Cell Signaling Technology) overnight at 4 °C and a secondary antibody (Cell Signaling Technology) for 1 h at RT. Finally, the membrane was visualized using a chemiluminescence method. All blots or gels derive from the same experiment and they were processed in parallel.

### Statistical analysis

Statistical analyses were performed by blinded researchers with GraphPad Prism 9.0 (GraphPad Software, San Diego, CA). Data were analyzed with two-way ANOVA to evaluate interactions among the groups, and Tukey’s test was used to detect differences between groups and the repeated measures. One-way ANOVA followed by Tukey’s post hoc test was used for experiments with more than two groups. Independent-sample t*-*tests were used to analyze all other data. Statistical significance was set to *p* < 0.05.

### Supplementary information

Supplementary Information file

Related Manuscript File

## Data Availability

The data that support the findings of this study are available from the corresponding author upon reasonable request.
