# Supplementary material for: Phosphorylation of AQP4 by LRRK2 R1441G impairs glymphatic clearance of IFNγ and aggravates dopaminergic neurodegeneration
Source: NPJ Parkinsons Dis. 2024 Jan 31;10:31. doi: 10.1038/s41531-024-00643-z (PMC10831045; doi:10.1038/s41531-024-00643-z)

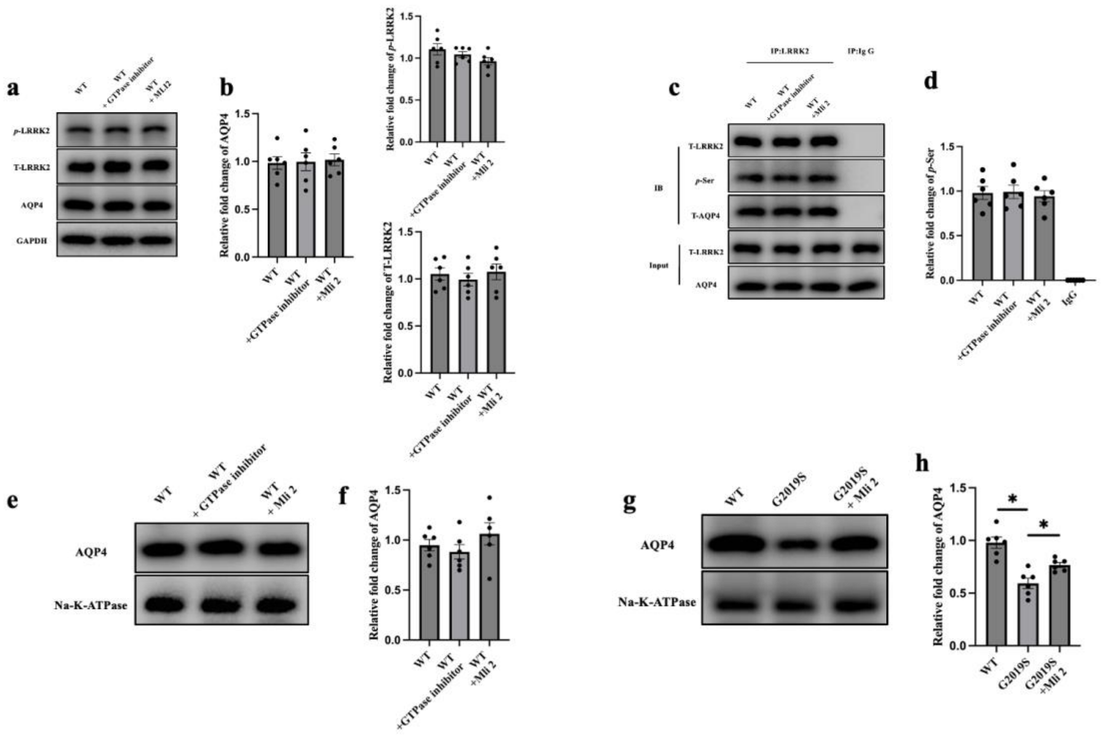

Supplementary Figure 1. LRRK2 interacts with AQP4 and phosphorylates AQP4. (a) Immunoblotting of proteins from brain lysates of the *LRRK2* WT transgenic mice treated with or without a GTPase inhibitor/Mli 2. **(b)** Quantification of a. Datasets are expressed as means ± standard error of mean. n = 6 per group. One-way ANOVA and Tukey's post hoc test were used for analysis. **(c)** Immunoblotting of phosphorylated serine (*p*-Ser) in AQP4 pulled down by LRRK2 from brain lysates of the *LRRK2* WT transgenic mice treated with or without a GTPase inhibitor/Mli 2. (**d**) Quantification of c. Datasets are expressed as means ± standard error of mean. n = 6 per group. One-way ANOVA and Tukey's post hoc test were used for analysis. **(e)** Immunoblotting of membrane proteins from brain lysates of *LRRK2* WT transgenic mice treated with or without a GTPase inhibitor/Mli 2. **(f)** Quantification of e. Datasets are expressed as means ± standard error of mean. n = 6 per group. One-way ANOVA and Tukey's post hoc test were used for analysis. **(g)** Immunoblotting of membrane proteins from brain lysates of *LRRK2* WT and *G2019S* transgenic mice treated with or without Mli 2. **(h)** Quantification of g. Datasets are expressed as means ± standard error of mean. n = 6 per group. *$p < 0.05$. Two-way ANOVA and Tukey's post hoc test were used for analysis.

Figure 2a

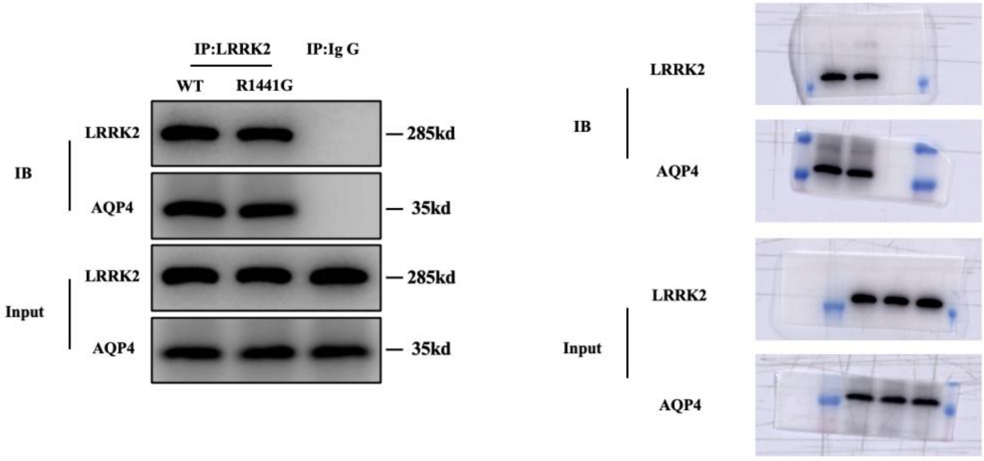

Figure 2c

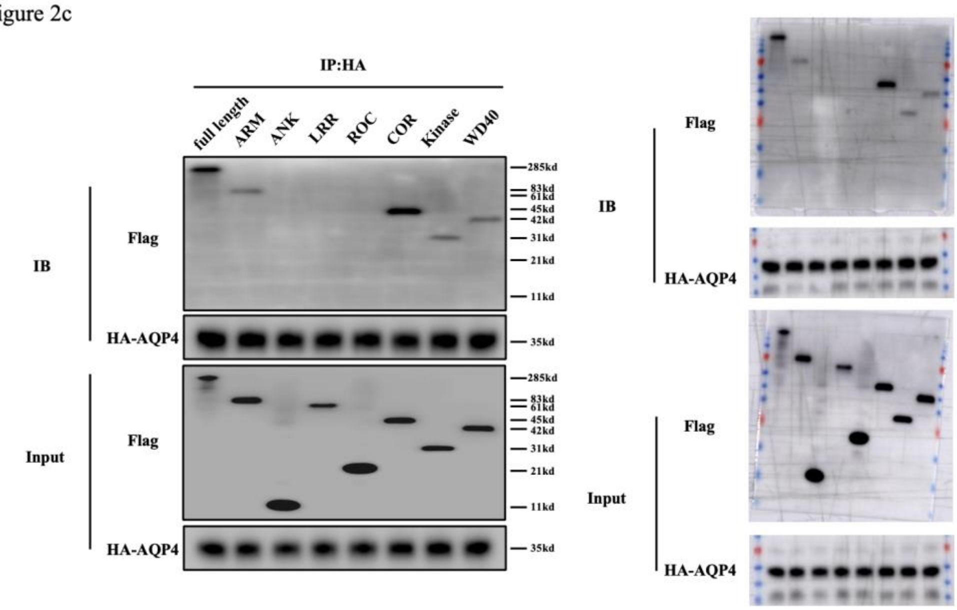

Figure 2d

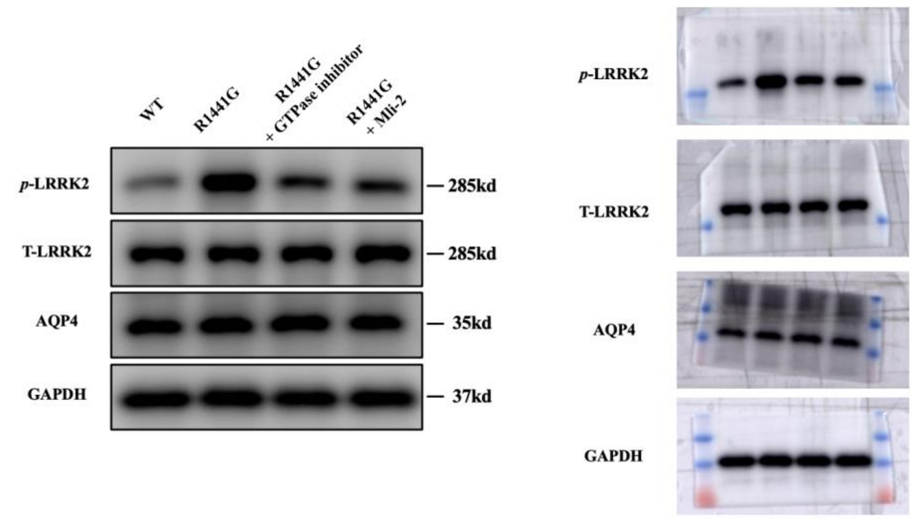

Figure 2f

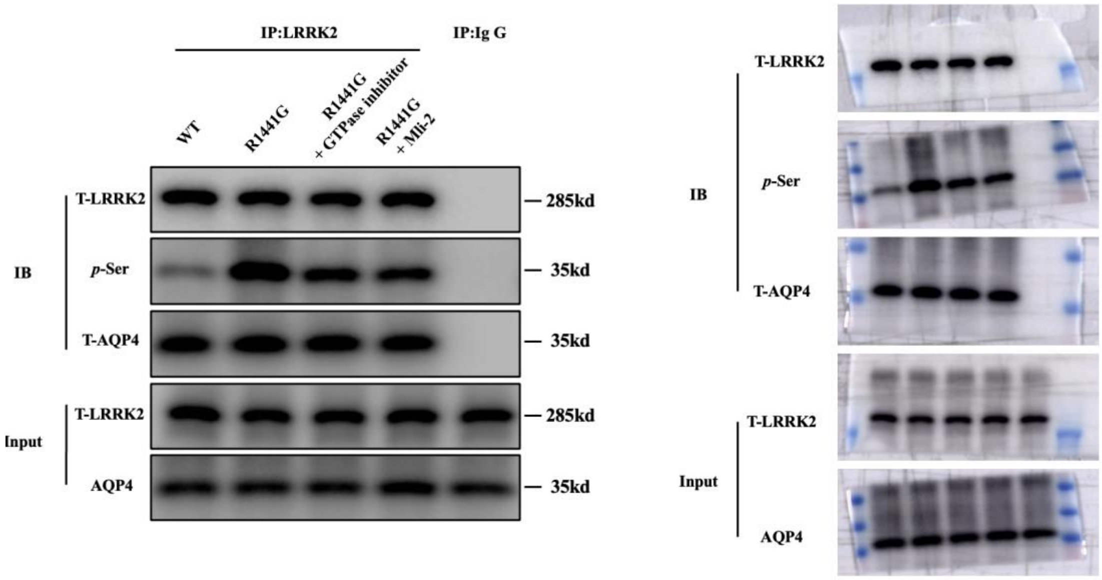

Figure 2h

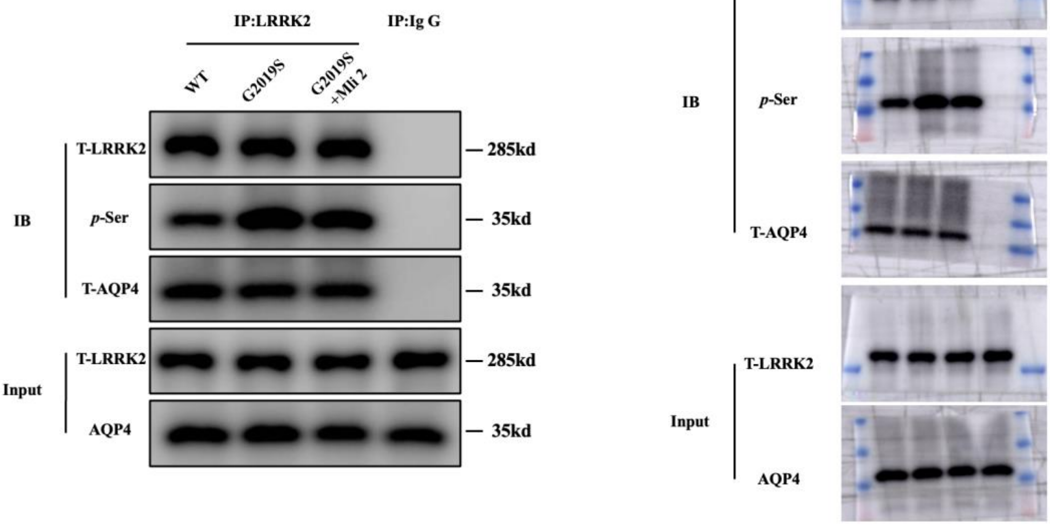

Figure 3e

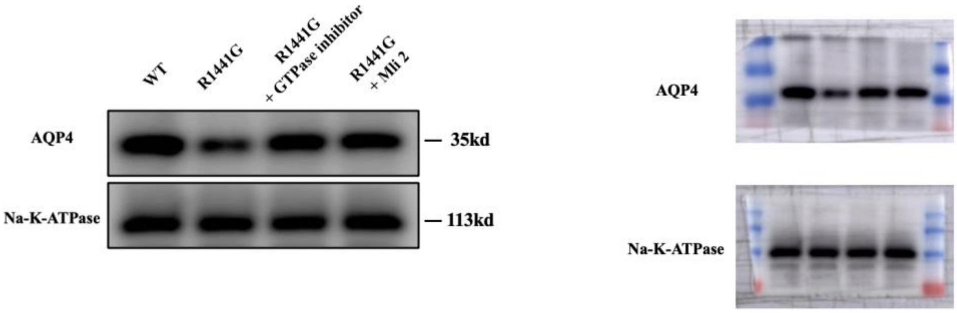

Supplementary Figure 1a

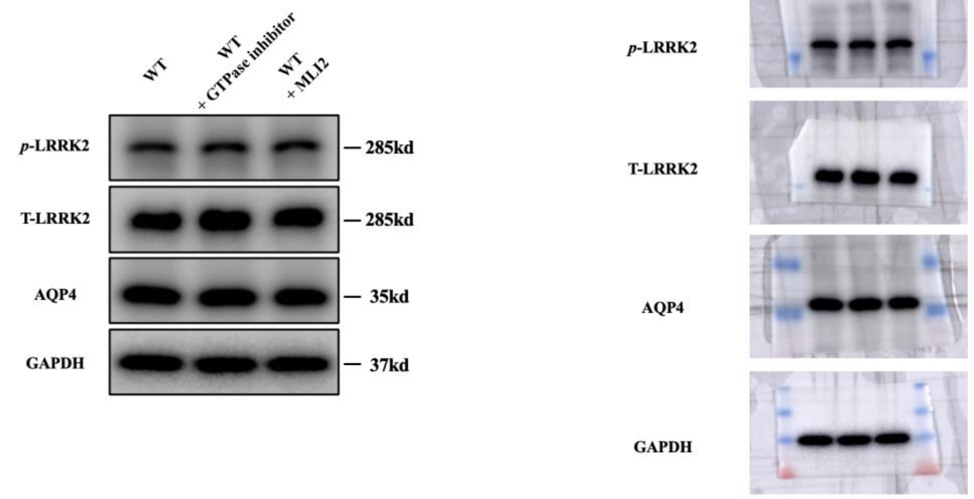

Supplementary Figure 1c

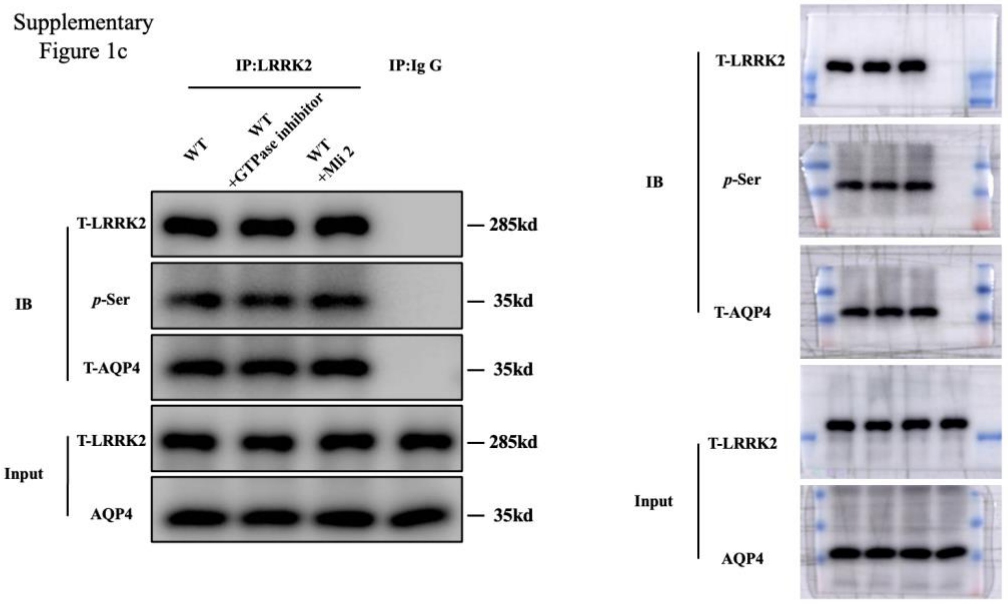

Supplementary
Figure 1e

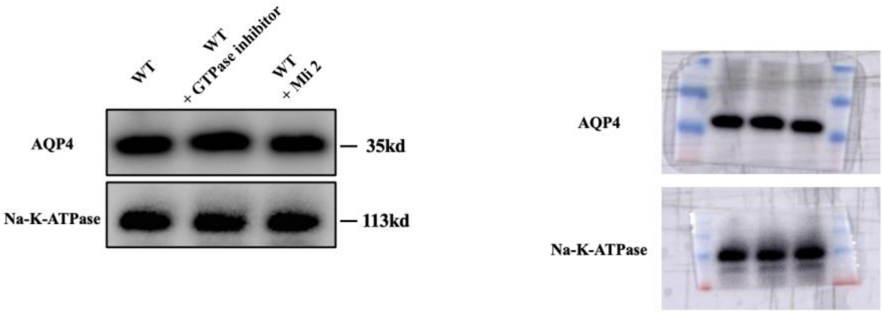

Supplementary
Figure 1g

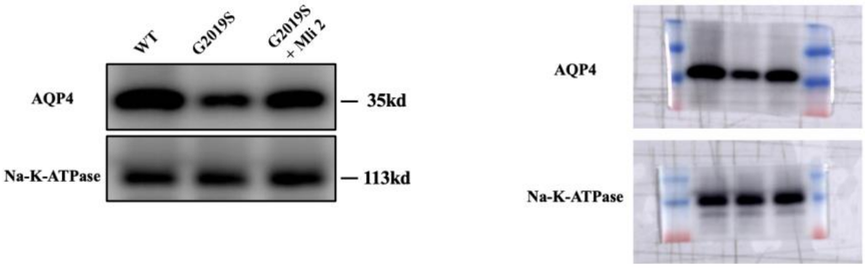

Figure 4b

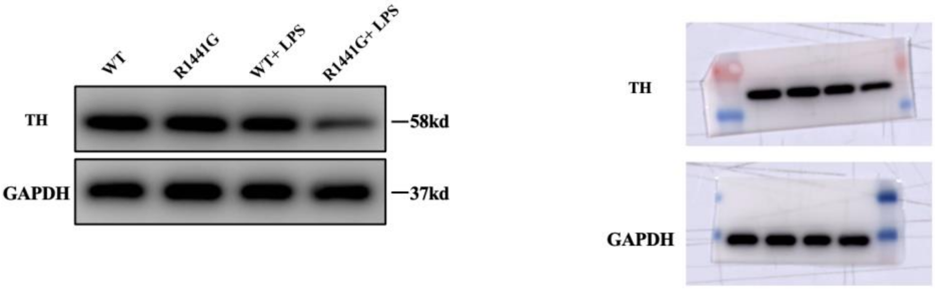

Figure 4d

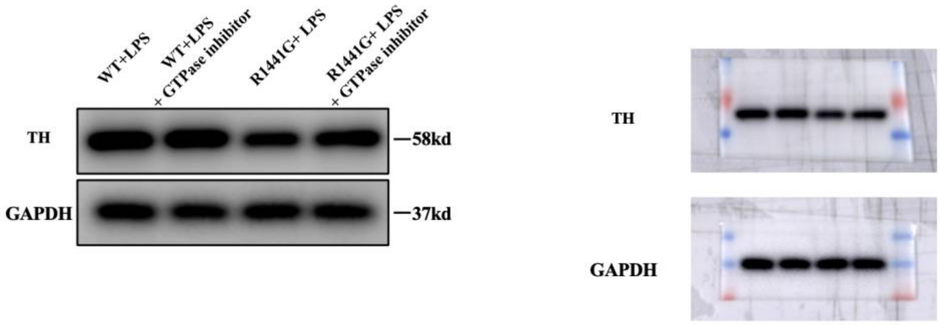

Figure 4i

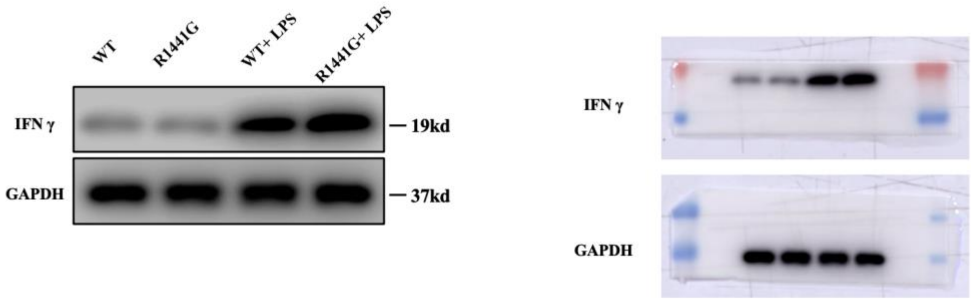

Figure 4k

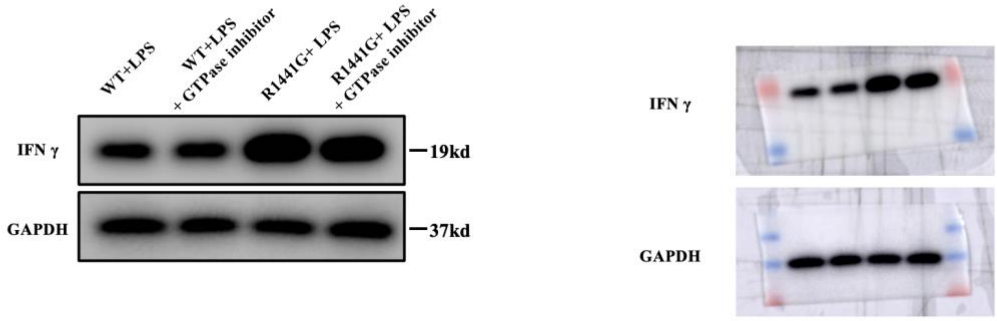

Figure 6a

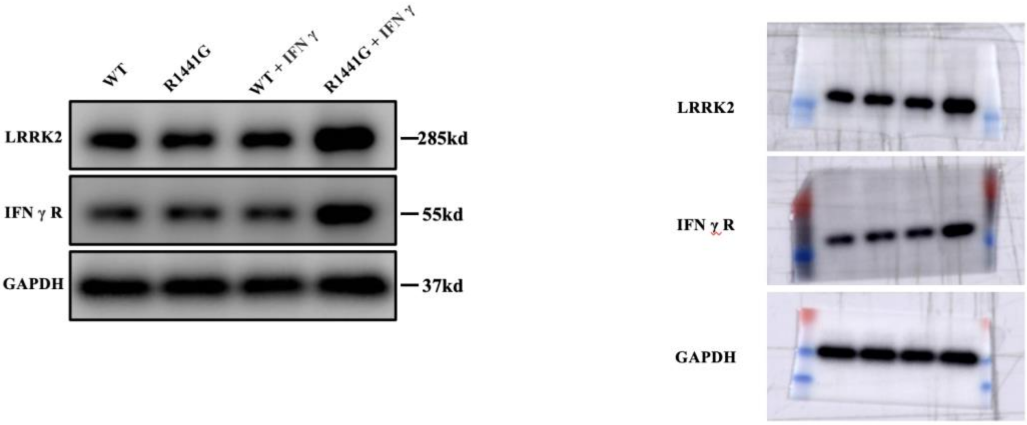

Figure 6d

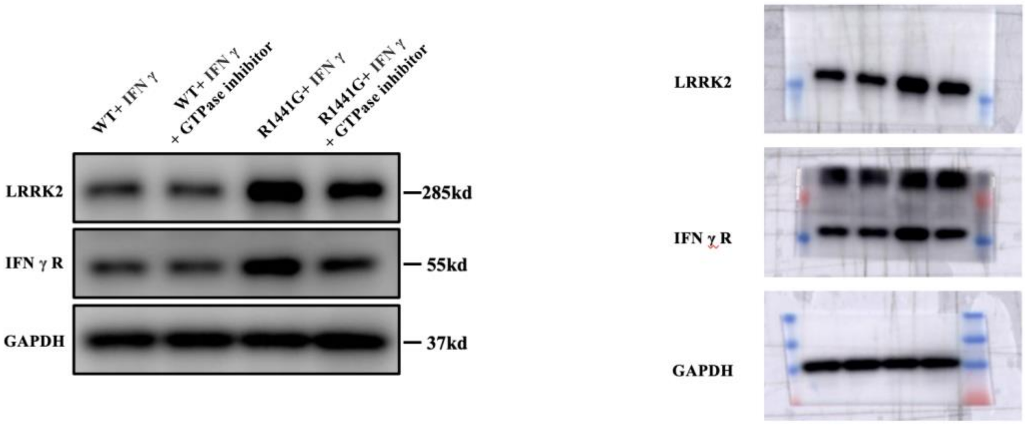

Figure 6k

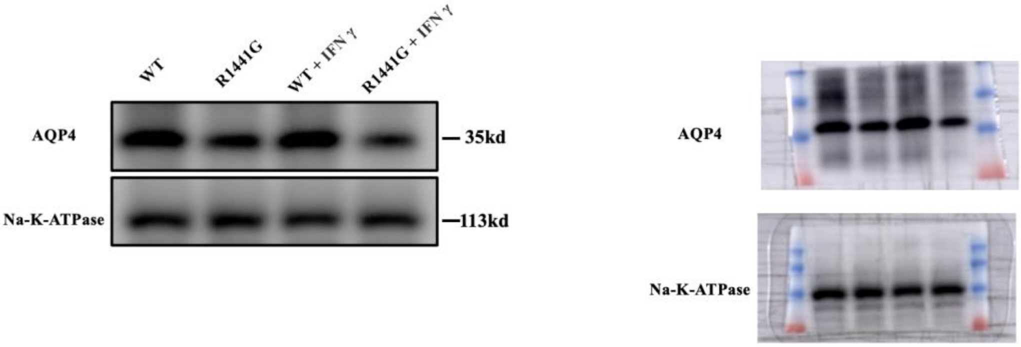

Figure 6m

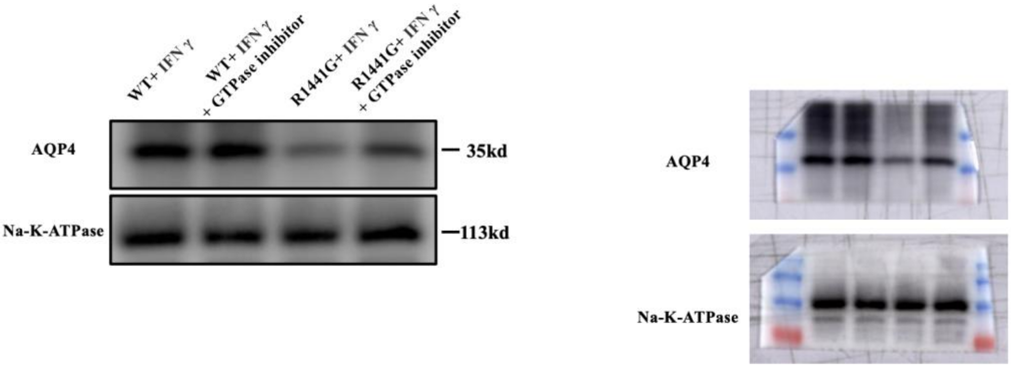

Figure 6o

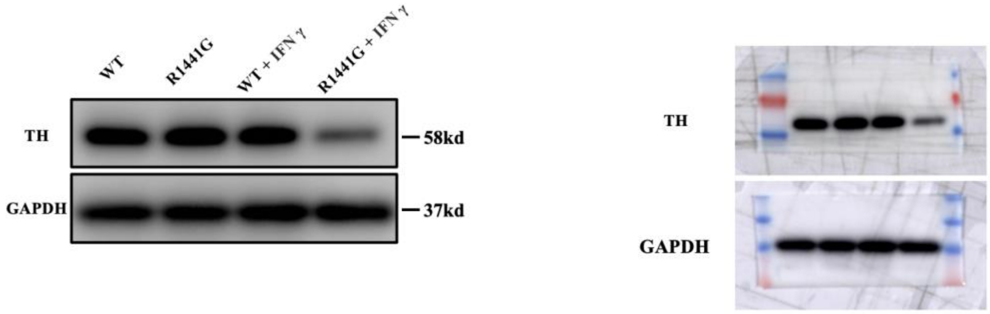

Figure 6q

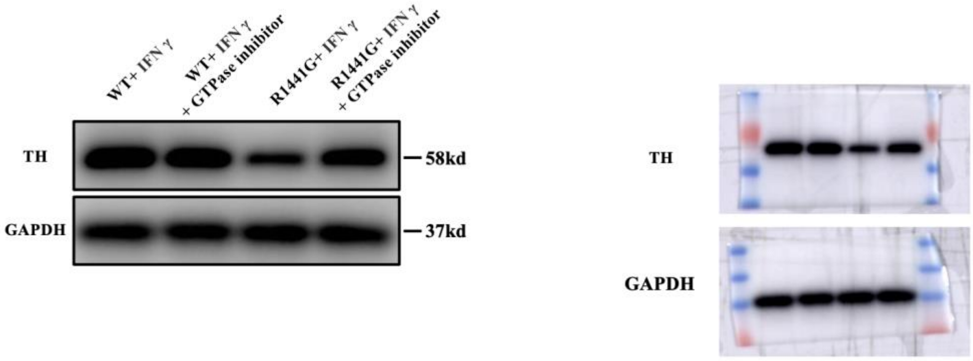

Supplement: Supplementary file 1 — Supplementary Information file [file 41531_2024_643_MOESM1_ESM.pdf]
